# Analysis of the sense of occupational stress and burnout syndrome among physiotherapists during the COVID-19 pandemic

**DOI:** 10.1038/s41598-023-32958-x

**Published:** 2023-04-07

**Authors:** Dorota Wójtowicz, Joanna Kowalska

**Affiliations:** Faculty of Physiotherapy, Wroclaw University of Health and Sport Sciences, Paderewskiego 35 Street, 51-612 Wrocław, Poland

**Keywords:** Psychology, Diseases, Health care, Health occupations

## Abstract

The nature of physiotherapists’ work involves an increased risk of occupational stress and burnout, particularly during the COVID-19 pandemic. Therefore, the aim of the study was to analyse the level of perceived generalised stress, the occupational stress and the occupational burnout syndrome among physiotherapists during the COVID-19 pandemic. One hundred seventy professionally active physiotherapists participated in the study: 100—during the pandemic and 70 before the COVID-19 pandemic. The study was carried out using the authors’ survey, the Subjective Work Assessment Questionnaire (SWAQ), the Oldenburg Burnout Inventory (OLBI), the Perceived Stress Scale (PSS-10), and the Brief Coping Orientation to Problems Experienced (Mini-COPE) inventory. The physiotherapists examined prior to the pandemic exhibited a higher level of generalised stress and higher level of occupational stress and occupational burnout (*p* = 0.0342; *p* < 0.00001; *p* < 0.00001, respectively). The key factors which caused intensified occupational stress in both groups included the lack of rewards at work, social interaction, and the lack of support. The results suggest that healthcare professionals including physiotherapists are exposed to occupational stress and a high risk of occupational burnout, not only during the COVID-19 pandemic. Occupational stress prevention programmes should be based on the identification and elimination of all occupational risks.

## Introduction

During the COVID-19 pandemic, declared by the World Health Organisation (WHO) on 11 March 2020, it was observed that due to having found themselves in an unexpected and unfamiliar situation, many people experienced increased stress. Taking care of one's own mental health and proper psychosocial functioning at that time became just as important as taking care of physical health^1^.

Researchers have observed an increased risk of occupational stress and burnout among healthcare professionals, including physiotherapists^2–7^. They constitute a high-risk group^8^, since they are exposed to occupational and emotional stress caused primarily by continuous contact with individuals who require their help and assistance. The sense of responsibility for the patients’ life and health generates excessive stress. Rasmus et al. and other researchers have demonstrated that occupational burnout can affect also very young individuals, including students majoring in medical fields^9–12^.

Naushad et al. have established that disasters have a negative impact on the mental wellbeing of paramedics. The most important factors in all types of disasters, which had the most dramatic negative impact, included the lack of society's support and effective communication, inadequate coping methods, and the lack of training^13^.

The existing studies suggest that the COVID-19 pandemic has had profound effects on the mental health states of healthcare workers^14,15^. The healthcare professionals were more exposed to stress^16,17^. Those workers were the primary individuals/specialists involved in the screening for and treatment of COVID-19 ^18^. At that time, multiple stress-inducing factors emerged, including the risk of infection, the lack of adequate protection against contamination, overwork, frustration, discrimination, isolation, patients experiencing negative emotions, the lack of contact with family, and exhaustion^19,20^. The difficult situation of healthcare professionals in Wuhan, who worked under tremendous pressure at the beginning of the pandemic, gave rise to numerous problems related to mental health, such as stress, anxiety, symptoms of depression, insomnia, denial, anger, and fear. In turn, those issues can cause distraction, disturb the thought process and impair the ability to take decisions which are crucial for the treatment of COVID-19 patients; furthermore, they can also have a permanent effect on the general wellbeing of healthcare professionals^21^. Research conducted by Liu et al. in China showed that healthcare professionals who worked in hospitals experienced symptoms of anxiety. They were particularly visible in those workers who were in direct contact with infected patients^22^.

Furthermore, the examinations of the level of occupational stress and burnout among physiotherapists during the COVID-19 pandemic confirm that they experience more severe generalised stress, which has been observed e.g. among Brazilian physiotherapists^5^. Anxiety and depression have also been reported in physiotherapists from South Korea. Therefore, attention is being drawn to the mental condition of physiotherapists, which should be continuously and carefully monitored^16^, while the prevention should focus on designing coping strategies for both physical therapists and healthcare professionals in general. It is therefore necessary to recognise stress-inducing factors at the workplace by conducting extensive research also under the unique circumstances of the ongoing pandemic.

The aim of the study was to analyse the level of perceived generalised stress, the level of occupational stress, the occupational burnout syndrome, and the coping strategies among physiotherapists during the COVID-19 pandemic and to compare those results with the data obtained prior to the pandemic. The hypothesis was that the mental condition of the physiotherapists examined during the pandemic would be worse than that of the physiotherapists examined prior to the pandemic.

## Materials and methods

### Participants

The pilot observational study was conducted in the Lower Silesian Voivodeship. The respondents were selected based on the non-random snowball sampling model, in which participants recruit other participants for the study.

The cross-sectional study consisted of 170 professionally active physiotherapists, of whom 100 were examined during the COVID-19 pandemic (between January and May 2021). The obtained data were compared with the results of the examinations carried out prior to the pandemic (70 physiotherapists examined between November 2019 and February 2020) by Kowalska et al.^11^ and published in *Medicina 2021*. Both groups were similar in terms of sex, length of professional experience and involvement in extra work, but they differed significantly in terms of age, workplace, education, and marital status. The detailed data are presented in Table 1.

**Table 1:** Characteristics of two study groups (Student's *t*-test and χ^2^ test).

Characteristic	Pre-COVID data N = 70		COVID-19 data N = 100		*p* (Student’s *t*-test)	Effect size
	M	SD	M	SD		*Hedges’ g*
Age	40.1	11.6	31.9	8.0	< 0.00001	0.85
Length of professional experience	13.0	8.0	12.7	5.3	NS	0.04
Sex	n	%	N	%	*p* (χ2)	*Cramér's V*
Female	56	80	70	70	NS	0.11
Male	14	20	30	30		
Workplace						
Sanatorium/hospital	51	73	16	16	< 0.00001**	0.57
Private outpatient clinic	12	17	60	60		
Public outpatient clinic	7	10	24	24		
Marital status						
Single/divorced/widowed	24	34	56	56		0.21
Married/in a relationship	46	66	44	44	0.0052**	
Education						
Physiotherapy technician	19	27	4	4		0.40
BSc in physiotherapy	23	33	20	20	< 0.00001**	
MSc in physiotherapy	28	40	76	76		
Involvement in extra work						
Yes	19	27	26	26		0.01
No	51	73	74	74	NS	
Work on saturdays and sundays						
Yes	36	51	8	8		0.58
No	13	19	74	74	< 0.00001**	
Occasionally	21	30	18	18		

*BSc* Bachelor's degree in physiotherapy, *MSc* Master in physiotherapy, *NS* not statistically significant values.

**Statistically significant values *p* < 0.05.

### Ethical statements

The research procedures were approved by the Senate Commission for the Ethics of Scientific Research of the Wroclaw University of Health and Sport Sciences, and with the Declaration of Helsinki.

The study was conducted in the form of anonymous questionnaires, without any intervention or experiment, with the informed consent of all participants. It was carried out under the supervision of the Faculty of Physiotherapy of the Wroclaw University of Health and Sport Sciences. In addition, all methods were performed in accordance with the relevant guidelines and regulations.

### Measurement

The study was carried out using the Subjective Work Assessment Questionnaire (SWAQ), the Oldenburg Burnout Inventory (OLBI), the Perceived Stress Scale (PSS-10) and the Brief Coping Orientation to Problems Experienced (Mini-COPE) inventory^11^. Sociodemographic data (e.g. age, sex, marital status, education) and information concerning the participants’ work (e.g. workplace, involvement in extra work, work on Saturdays and Sundays) were collected using the authors’ own survey. That survey also included questions concerning work during the COVID-19 pandemic (e.g. being in quarantine, testing for COVID-19, getting sick with COVID-19, presence of patients infected with COVID-19 at the workplace, reduction of working hours due to the pandemic, fewer or no more home visits due to the pandemic).

The SWAQ, developed by Dudek et al. evaluates the stress and identifies the most stress-inducing psychosocial and physical factors present at the workplace. It consists of 57 questions which address various characteristics of work, classified into ten factors: sense of mental strain associated with the complexity of work, lack of rewards at work, sense of uncertainty resulting from workplace organisation, social interaction, sense of hazard, physical inconvenience, unpleasant working conditions, lack of control, lack of support, and the sense of responsibility. The participants answered the questions using a scale from 1 to 5 (1—the absence of the given factor; 5—highly onerous nature of the given factor). The higher the total score, the more intense the occupational stress. The points given to individual factors, compared with the values included in the standards table compiled by the authors of the SWAQ, indicate the categories of factors which pose a particular hazard to the employee. The Cronbach’s alpha for the questionnaire as a whole is 0.84^23^.

The OLBI created by Demerouti and adapted in Polish by Chirkowska-Smolak evaluates the level of occupational burnout in professionally active individuals. It consists of two subscales—exhaustion and disengagement (cynicism). There are 16 questions, 8 for each subscale. The higher the score, the higher the level of occupational burnout. The Cronbach’s alpha for the scale as a whole was above 0.7, and amounted to 0.75 and 0.47 for the exhaustion and the disengagement subscales, respectively^24,25^.

The PSS-10, created by Cohen et al. and adapted in Polish by Juczyński and Ogińska-Bulik, consists of 10 questions and measures the subjective perceived stress and feelings associated with stressful situations. The higher the score (max. 40 points), the higher the intensity of perceived stress. The raw score is interpreted using sten scores. A sten score of 1–4 (0–13 points) is considered low, a sten score of 5–6 (14–19 points) is considered moderate, and a sten score of 7–10 (20–40 points) is considered high. The Cronbach’s alpha for this scale was 0.86^26^.

The Mini-COPE inventory, developed by Carver and adapted in Polish by Juczyński and Ogińska-Bulik, is used to evaluate the typical coping strategies and reactions in situations where the individual experiences severe stress. It consists of 28 statements which refer to 14 strategies describing various coping styles. The higher the score, the more frequently the given strategy is employed by the participant. Strategies such as active coping, planning, and use of instrumental support are referred to as “problem-focused”. Use of emotional support, religion, or denial are considered “emotion-focused”. Venting, self-distraction, behavioural disengagement, substance use, and humour signify avoidance; nevertheless, they can bring short-term relief. Split-half reliability was 0.86 (Guttman split-half coefficient—0.87)^26^.

### Statistical analysis

The measures of descriptive statistics such as mean, standard deviation and in the case of qualitative variables percentages and amounts were used. The normality of distribution was verified using the Kolmogorov–Smirnov test. Due to the normality of the distribution of variables, the significance of differences between the groups was verified using the Student’s *t*-test and the *χ*^*2*^ test. Furthermore, in order to determine the effect size of differences between the study groups, Hedges’ g (due to the different sample sizes) and Cramér’s V were used. The assumed significance level was *p* < 0.05.

## Results

Only 28% of the examined physiotherapists had contracted COVID-19 and 51% had undergone at least one COVID-19 test. The majority of the study participants (59%) reported that patients infected with COVID-19 were present at their workplace and 23% ceased to offer home visits due to the pandemic (Table 2).

**Table 2:** Responses given by the examined physiotherapists to selected questions from the survey concerning the COVID-19 pandemic.

Selected questions from the survey	N (%)
Quarantine	
Yes	34 (34)
No	66 (66)
COVID-19 test	
Yes	51 (51)
No	49 (49)
Personal history of COVID-19	
Yes	28 (28)
No	72 (72)
Presence of patients infected with COVID-19 at the workplace	
Yes	59 (59)
No	41 (41)
Reduction of working hours due to the pandemic	
Yes	24 (24)
No	76 (76)
Reduction of the salary due to the pandemic	
Yes	19 (19)
No	81 (81)
Fewer or no more home visits due to the pandemic	
Yes	23 (23)
No	19 (19)
Not applicable	58 (58)

The comparative analysis of the results from both groups showed a statistically significant difference in the level of occupational stress, the level of perceived generalised stress, and the level of occupational burnout (Table 3).

**Table 3:** Comparison of the results obtained prior to and during the COVID-19 pandemic (independent samples Student's *t*-test).

Scales	Pre-COVID data N = 70		COVID-19 data N = 100		t	*p*	Effect size
	M	SD	M	SD			*Hedges’ g*
SWAQ total (sten)	7.63	1.9	5.73	2.2	5.88	< 0.00001**	0.91
PSS-10 (sten)	5.99	1.9	5.38	1.7	2.13	0.0342**	1.26
OLBI—Exhaustion (sten)	5.97	1.6	4.68	1.8	4.76	< 0.00001**	0.75
OLBI—Disengagement (sten)	5.83	1.7	4.36	1.8	5.24	< 0.00001**	0.83

*SWAQ* the Subjective Work Assessment Questionnaire, *PSS-10* the Perceived Stress Scale, *OLBI* the Oldenburg Burnout Inventory.

**Statistically significant values *p* < 0.05.

The qualitative analysis also demonstrated a significantly larger number of cases of a high level of occupational stress in the group of physiotherapists examined prior to the pandemic (Table 4).

**Table 4:** Comparison of the PSS-10 and SWAQ results—a qualitative analysis (*χ*^*2*^* test)*.

	Pre-COVID-19 data N = 70		COVID-19 data N = 100		*χ*^*2*^	*p*	Effect size Cramér's V
	n	%	n	%			
SWAQ results							
Low (sten score of 1–4)	5	7	29	29			
Moderate (sten score of 5–6)	14	20	32	32	20.9	0.00003**	0.35
High (sten score of 7–10)	51	73	39	39			
PSS-10 results							
Low	16	23	37	37			
Moderate	26	37	28	28	4.0	0.1351	0.15
High	28	40	35	35			

*SWAQ* the Subjective Work Assessment Questionnaire, *PSS-10* the Perceived Stress Scale.

**Statistically significant values *p* < 0.05.

The most significant factors which influenced the total score of the workplace stress intensity in the group of physiotherapists examined during the pandemic included the lack of rewards at work, social interaction, and the lack of support. Statistically significant differences were found between the groups in respect of all factors included in the SWAQ scale, except for *Lack of support* (Table 5).

**Table 5:** The SWAQ results prior to and during the COVID-19 pandemic (independent samples Student's *t*-test).

SWAQ	Pre-COVID-19 data N = 70		COVID-19 dataN = 100		t	*p*	Effect size *Hedges’ g*
	M	SD	M	SD			
SWAQ total score (sten)	7.63	1.9	5.73	2.2	5.88	< 0.00001**	0.91
Sense of mental strain associated with the complexity of work	16.9	6.2	14.9	5.3	2.19	0.0296**	0.35
Lack of rewards at work	18.2*	6.4	14.0*	6.3	4.24	0.00004**	0.66
Sense of uncertainty resulting from workplace organisation	17.3*	5.7	12.8	5.0	5.42	< 0.00001**	0.85
Social interaction	9.9*	3.1	9.1*	2.4	1.99	0.0475**	0.29
Sense of hazard	10.9*	3.4	8.8	3.1	4.28	0.00003**	0.65
Physical inconvenience	6.8	3.2	1.5	1.0	15.56	< 0.00001**	2.42
Unpleasant working conditions	5.1*	2.3	1.4	1.0	14.13	< 0.00001**	2.23
Lack of control	9.6*	2.6	7.8	2.3	4.53	0.00001**	0.74
Lack of support	5.4*	2.2	5.0*	2.5	1.21	0.2272	0.17
Sense of responsibility	8.3*	2.9	7.1	2.9	2.70	0.0075**	0.41

*SWAQ* the Subjective work assessment questionnaire.

*Highly stress-inducing factors.

**Statistically significant values *p* < 0.05.

While experiencing severe stress, the physiotherapists examined during the pandemic most often employed the following strategies: planning, active coping, and use of emotional support, similarly to the physiotherapists examined prior to the pandemic (Table 6).

**Table 6:** Mini-COPE results prior to and during the COVID-19 pandemic.

Mini-COPE	Pre-COVID-19 data N = 70		COVID-19 data N = 100	
	M	SD	M	SD
1. Active coping	2.1*	0.5	2.0*	0.7
2. Planning	2.1*	0.5	2.1*	0.7
3. Positive reframing	1.8	0.6	1.8	0.8
4. Acceptance	1.8	0.6	1.7	0.7
5. Humour	1.0	0.7	1.2	1.6
6. Religion	1.1	1.0	0.9	1.0
7. Use of emotional support	2.0*	0.7	1.9*	0.8
8. Use of instrumental support	1.8	0.6	1.8	0.7
9. Self-distraction	1.6	0.7	1.5	0.7
10. Denial	0.8	0.7	0.6	0.6
11. Venting	1.5	0.6	1.4	0.7
12. Substance use	0.5	0.8	0.4	0.6
13. Behavioural disengagement	0.8	0.6	0.7	0.7
14. Self-blame	1.0	0.6	1.3	0.8

Mini-COPE – the Brief Coping Orientation to Problems Experienced inventory.

*The most commonly employed strategies.

## Discussion

Kang et al. and Chirico et al. have established that both healthcare professionals and other workers experienced mental stress during the pandemic and the mental problems of healthcare professionals were more severe and required more attention and mental support in order to improve the general mental condition of those affected. This knowledge focused the attention worldwide on the need to take parallel actions, i.e. reduce the spread of COVID-19 and conduct research on the impact of this disease on mental functioning^14,21,27,28^.

Research conducted by Kowalska et al.^11^ prior to the pandemic demonstrated that the greater exposure of physiotherapists to psychosocial occupational risks causes a more severe sense of generalised stress and a higher level of occupational burnout. By comparing two groups, this article strove to explain the functioning of physiotherapists under diverse working conditions (during the pandemic), with the assumption—based on the existing research—that the mental condition of the physiotherapists examined during the pandemic would be worse than that of the physiotherapists examined prior to the pandemic. However, the results did not support this hypothesis. A significantly higher level of generalised stress, a significantly higher level of occupational stress, as well as a significantly higher level of occupational burnout were indeed found, but in the group of the physiotherapists examined prior to the COVID-19 pandemic. These results were also confirmed by the qualitative analysis, in particular of the occupational stress, where a higher number of cases of a high level of occupational stress was found among the physiotherapists examined prior to the pandemic. Similar results were recorded in respect of nearly all stress-inducing factors included in the SWAQ scale. The *Lack of support* factor was an exception—in addition to *Lack of rewards at work* and *Social interaction*, it was the most stress-inducing factor in both study groups. The results are surprising, but after a thorough analysis of both groups of participants, certain aspects can be consistent with other reports. First and foremost, only 28% of the physiotherapists examined during the pandemic contracted the disease and 34% of them had been quarantined. These percentages are low and may have affected the results of the study but an Italian study conducted during the COVID-19 pandemic showed that healthcare workers who were not infected experienced the same high levels of stress^15^. This can be attributed to the type of workplace, which represented a significant difference between the groups. This group significantly more often worked in the private sector, similarly to the study of Portuguese physiotherapists, but with different results for the occupational burnout^29^. Therefore, despite the ongoing pandemic, the working conditions may have been better and the contact with infected patients—less frequent, which is why the presence of infected patients at the workplace was reported by 59% of the physiotherapists. Moreover, further analysis of the data describing both groups demonstrated that additional significant differences between the groups were observed in respect of age, education, marital status, and working on Saturdays and Sundays. The physiotherapists examined during the pandemic were approximately 8 years younger than the physiotherapists examined prior to the pandemic. In this group, a significantly larger number of physiotherapists held a master’s degree, was single, divorced or widowed, and did not work on Saturdays or Sundays. The coincidence of characteristics of this group may have had a significant influence on the lower level of generalised stress, occupational burnout and the level of occupational stress during the pandemic. Research conducted by Pustułka-Piwnik et al. also demonstrated that the level of occupational burnout among physiotherapists are significantly related to the selected demographic and organisational variables^30^.

The studies carried out in South Korea during the COVID-19 pandemic showed that in terms of age, none of the examined physiotherapists aged 20 suffered from depression and the risk of that disorder among the individuals aged 30 and 50 was markedly higher. In addition to the relationship between the physiotherapist’s age and the incidence of depression, it was observed that the life situation, e.g. living with a child aged 0–6, increased the likelihood of anxiety^16^.

Different results, obtained during the study on healthcare professionals working in hospitals under quarantine in Egypt, were presented by Youssef et al., who emphasised that younger healthcare professionals were more likely to report adverse mental symptoms^4^. Further studies on primary healthcare workers in Egypt showed that a significant percentage of the participants experienced symptoms of anxiety, insomnia, depression and stress^31^.

It should be taken into consideration that long-term exposure of physiotherapists to highly stress-inducing factors at the workplace can have a significant impact on the progress of occupational burnout. A relationship between occupational stress and occupational burnout was demonstrated, which confirms that the accumulated effect of stress can lead to burnout^32^. The existing reports on occupational burnout among physiotherapists in Poland and worldwide are varied, including those pertaining to the pandemic. In contrast to our results, Pniak et al. emphasise that the incidence of burnout among physiotherapists may have significantly increased during the COVID-19 pandemic. However, given the scarcity of scientific evidence related to this specific problem in Poland and worldwide, it is necessary to continue research into occupational burnout which affects physiotherapists^33^. Furthermore, Jácome et al. have noticed that while comparing the data with international studies focusing on physiotherapists, it can be observed that slightly lower burnout levels have been reported prior to the COVID-19 pandemic. They emphasise that in fact the burnout level had already been alarming before the pandemic, with around ∼ 10 to 20% of physiotherapists experiencing high level of burnout and with ∼ 30 to 50% facing a high risk of developing it^29^. This was confirmed also during our research. The already existing, inadequate working conditions, the lack of time, overwork and occupational burnout among physiotherapists may have an adverse impact not only on the effectiveness of the treatment, but also on the quality of life of both the patients and the physiotherapists^3^. The pandemic may have additionally exacerbated the inadequate working conditions faced by physiotherapists.

Both prior to and during the pandemic, the dominant coping strategies employed by the examined physiotherapists included active coping, planning, and use of emotional support. As active strategies, active coping and planning are associated with a lower sense of stress. In turn, the use of emotional support is a strategy focused on emotions. It can be a sign of adaptation, but this is not clear^26^. Psychological assistance and support groups should therefore be standard elements of mental healthcare of physiotherapists who work in various conditions and circumstances. The more, so psychological interventions and psychological support programs in the healthcare sector are effective and cost-saving tools^34^.

Additionally, the occupational health surveillance and workplace health promotion programmes for early prevention of mental disorders and promotion of high level of mental well-being are needed^20^. It is possible in the times post-COVID- 19 pandemic through a strict cooperation between public health and occupational health stakeholders^35,36^.

Occupational stress prevention programmes should be based on the identification and elimination of all occupational risks, including the psychosocial factors at the workplace.

The differences in the functioning of the healthcare system, salaries, the economic situation, and the research methodologies employed by various authors make the analysis of this issue more complicated. The pandemic poses yet another challenge for practitioners and researchers in terms of the thorough examination of this subject, especially since, according to Shigemura et al.^37^ the psychological consequences of a pandemic usually persist long after it ends.

Further research into the prevention of stress, occupational stress, and occupational burnout among physiotherapists is economically and socially justified, and will have a profound impact on the professional tasks performed by physiotherapists, i.e. functional diagnostics, physical therapy planning, implementation of treatment, as well as the monitoring and modification of physiotherapy activities based on the individual needs of the patients.

## Limitations

The present study had some limitations. The used tests were screening in nature. A main limitation of the study is not very large study group and the non-random sampling. Therefore, the results obtained should not be generalised. In future research, incorporating the measurement of stress biomarkers might be considered. The study was carried out at a single point in time. It does not show the dynamics of changes in the level of perceived generalised stress, the level of occupational stress, and the level of occupational burnout of the physiotherapists surveyed.

## Conclusions

1. A significantly higher level of generalised stress, occupational stress and occupational burnout was found in the group of physiotherapists examined prior to the pandemic as compared to the group of physiotherapists examined during the pandemic. As many as 73% of the participants examined prior to the pandemic were characterised by a high level of occupational stress.
2. The most significant factors which influenced the general level of occupational stress in both groups of physiotherapists included the lack of rewards at work, social interaction, and the lack of support. Nevertheless, the physiotherapists examined prior to the pandemic were characterised by significantly higher results in respect of all factors included in the SWAQ scale.
3. While experiencing severe stress, both groups of physiotherapists most often employed the following strategies: planning, active coping, and use of emotional support.
4. The results suggest that healthcare professional including physiotherapists are exposed to occupational stress and face a high risk of occupational burnout, not only during the COVID-19 pandemic.

## Data Availability

The datasets used and/or analysed during the current study available from the corresponding author on reasonable request.
